# Meeting Highlights: Joint Cold Spring Harbor Laboratory and Wellcome Trust Conference – Genome Informatics

**DOI:** 10.1002/cfg.114

**Published:** 2001-12

**Authors:** Jo Wixon, Jennifer Ashurst, Jo Dicks

**Affiliations:** 1 HGMP-RC Hinxton, Cambridge CB10 1SB UK; 2 Sanger Centre Wellcome Trust Genome Campus Hinxton, Cambridge UK; 3 John Innes Centre Norwich Research Park Colney, Norwich UK


**Comparative and Functional Genomics**
*Comp Funct Genom* 2001; **2**: 376–383.
**DOI:** 10.1002/cfg.114

**Meeting Highlights**

### Joint Cold Spring Harbor Laboratory and Wellcome Trust conference – genome informatics

Wellcome Trust Genome Campus, Hinxton, Cambridge, UK. August 8–12, 2001

Jo Wixon[1]*, Jennifer Ashurst[2] and Jo Dicks[3]
[1] *HGMP-RC, Hinxton, Cambridge, UK*
[2] *Sanger Centre, Wellcome Trust Genome Campus, Hinxton, Cambridge, UK*
[3] *John Innes Centre, Norwich Research Park, Colney, Norwich, UK*

**Correspondence to:*
*HGMP-RC, Hinxton,*
*Cambridge, CB10 1SB, UK.*
*E-mail: nh1@sanger.ac.uk*

Published online:
26 October 2001

### Annotation pipelines

Ewan Birney (EBI, UK) opened the meeting with a presentation on **the Ensembl gene building system (http://www.ensembl.org)**. The first part of the process is Exonerate (Guy Slater), this is the 'basic guts' of BLAST, allows multiplexing and makes a lazy evaluation of the path between HSPs, to rapidly build gapped alignments. This was designed for ESTs, but is now being applied to mouse whole genome shotgun data. The next stage is Pmatch (Richard Durbin), a hyper-fast protein-based exact matcher (similar to Jim Kent's BLAT). This finds exact 14mer substrings by building a table of non-overlapping 5mer matches, and using pairs of consecutive 5mer matches as seeds. A targeted gene build uses Pmatch to match all known human proteins to the entire genome, and is then refined using Genome Wise. Genome Wise (Ewan Birney) uses information such as EST alignments to the genome to build gene models (Figure 1 ). It can reconcile overlapping alignments and uses 'tunnelling' in the absence of splices in a match, this extends the ORF, where possible (until it finds a stop codon).

Tom Casavant (University of Iowa, USA) described **CAEPA, an online, bulk EST sequence processing and annotation pipeline**. The group's main aim is gene discovery using EST data, so far they have annotated and submitted ~500 000 ESTs. They have produced an automated initial annotation pipeline, and provide support for serial subtraction, which includes clustering to detect novelty, and large scale BLAST for synteny assessment. Their EST processing pipeline consists of an EST preparation stage with a vector and contaminant screen and a repeat masker and low complexity screen, a local annotation tool looks for 3′ and 5′

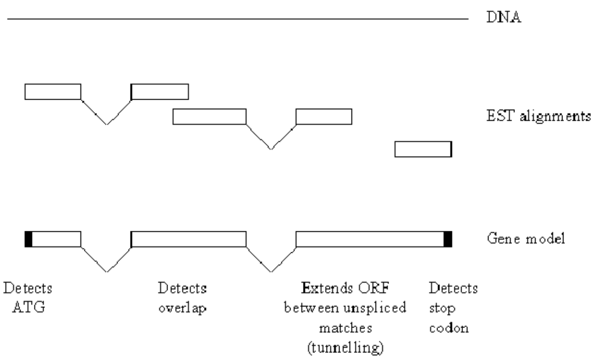

**Figure 1.** An illustration of the abilities of Genome Wise

Copyright © 2001 John Wiley & Sons, Ltd.

EST features, this is followed by clustering and then radiation hybrid mapping. To make this accessible to groups running small projects, they have produced a web 'front end' with a data drop point, and e-mail return of results from custom BLAST searches of internal datasets. Users can choose which parts of the pipeline they which to take advantage of and can make other selections such as which libraries of repeats to screen against.

Ron Chen (DoubleTwist, USA) described their strategy for **prioritising predicted human genes for experimental validation**. Their high-throughput approach starts with the *ab initio* gene prediction programs GenScan, FGENESH and GrailEXP. To this data they add protein homology data and results from comparisons to RefSeq, UniGene and their own gene index. These three sources of evidence are then considered by Gene Squasher, which makes locus predictions. It constructs exons by assembling overlapping candidates from the three methods and assembles them into genes, if there is a common gene model or other supporting evidence. All the data goes into their Agave XML format and they have a genomic viewer, which handles the data, showing the three types of exon data and the final predictions. Their current locus prediction counts are *ab initio* – 39 000, protein homology – 31 000, and gene index match – 68 000. Checking for overlaps brings the total down to ~98 000, half of these have only gene index match data, only 10% are detected by all three approaches. 30.2% (~29 500) are high confidence, with two lines of evidence.

William Fitzhugh (Whitehead Institute, USA) presented **Calhoun, a comprehensive system for genome sequence annotation**. A crucial part of this approach is that it has a platform to tie the existing stand-alone genome annotation tools together and keep track of results. It has a tool for viewing results and can manage and run a large number of jobs at any given time. Calhoun has an Oracle relational database, a Java sequence browser, web-based tools accessible by the public and an analysis pipeline. The database schema is huge, with a vast array of entities, from sequence, to taxa, to mapping data. They use SQL to ask biologically relevant questions. The system uses an analysis queue table to organise analysis jobs, which can keep track of all the jobs done on a particular sequence. Web 'front end' tools include a gene search and the ability to visualise the data and a combined physical and genetic map. Their current work is centred on *Neurospora crassa*, but they hope to scale up to a larger eukaryote genome next.

Inna Dubchak (LBNL, USA) described **a pipeline for real-time comparative analysis of the human and mouse genomes** (**http://pipeline.lbl.gov**), which is called Godzilla, because it is going to be huge. They download mouse data daily from GenBank and pass this through Masker Aid and a first round homology search using BLAT, to determine which sequences are then compared to the April freeze of the Golden Path human annotation using BLAT. The next stage is called AVID, this is a global alignment engine which can handle a combination of finished and draft sequence. The final alignments are viewed using VISTA. The pipeline has been used to discover conserved sequence fragments, which they have seen in coding and non-coding regions. It has also found overlapping contigs in GenBank and detected mis-assemblies. Their data is soon to be included in the UCSC genome viewer.

Michelle Clamp (EBI, UK) spoke about the **Ensembl analysis pipeline**. They currently have 4.3 Gb of data (480 000 sequence reads) to analyse. 16 types of analysis are used, which requires a lot of organisation. They have automated job submission, tracking programs, a set-up to retry failed jobs and access to large, file-based databases. They have a cluster with 2 Terabytes of storage and a farm of 320 machines with 60 Gb local drive. New entries go immediately into tracking, which determines which jobs need doing and what order to do them, then forms a job and sends it to the farm. Communication between the farm and the cluster is very complex, so they keep as much as possible on the local drives (hence their large size) such as BLAST databases, binaries and runtime files. The data is fetched or written directly to the databases. They use BioPerl (which Michelle likes very much) and MySQL. This is an open source project, the software and data are freely available. The entire system is portable and there are currently more than 10 remote installations of the website.

**GANESH; a sequence analysis and display package**, was presented by Holger Hummerich (Imperial College, UK). This is a user-friendly tool aimed at researchers interested in small regions, such as those hunting for disease genes (**http://zebrafish.doc.ic.ac.uk/Ganesh/ganesh.html**). It is dynamic and updates daily, allows selective use (such as choosing to view only new data), and can display data at varying levels of resolution, from clone contigs up to BLAST alignments. The system downloads the relevant data

Copyright © 2001 John Wiley & Sons, Ltd.

from GenBank and runs several analysis programs, including Repeat Masker, GenScan and Pfam. BLAST searches are performed across a wide range of databases. This open source tool uses a MySQL relational database and has a Java front-end display. The display shows the sequence across the centre of the window, with data for one strand above and the other strand below. Features such as ESTs, SNPs, repeats, exons and promoters are marked on the display in different colours and users can add their own annotation.

**TIGR gene indices (http://www.tigr.org/tdb/tgi.shtml)** have now been assembled for 13 animal species, 11 plant species, 12 protists and 5 fungi. Dan Lee (TIGR, USA) described how to build an index they take ESTs from GenBank and their own databases and remove vector, poly A tails, mitochondrial and ribosomal sequences etc. They then harvest expressed transcripts from GenBank cds entries and reduce the redundancy amongst these before comparing to the ESTs to build clusters. Each cluster is assembled into a tentative consensus (TC). TC reports contain the consensus sequence, a map of the ESTs in the cluster and details of each EST. All TCs are mapped back onto the genome in question. They have used a comparison of 28 indices to make **TOGA, the TIGR orthologous gene alignment database**. To assemble these they compare all genes in organism A against all genes in organism B, and then all genes in organism B against all genes in organism A, searching for reciprocal best matches (with $e^{-10}$). Bringing in more organisms enables them to build reciprocal best match networks (or clusters). Then they look for functional information about the members of the clusters. TOGA is being used to look at how pathways are (or are not) conserved across species, and for phylogenetic analysis.

### Genome assembly

Zemin Ning (The Sanger Centre, UK) spoke about **an ordering and orientation assembly of 3X mouse genome shotgun data**. This was done on 13.4 million reads, (including 5.6 million paired reads) totalling some 3 Gbp of sequence, and took 2.5 days. The end result was 1.5 million contigs, covering 1.6 Gbp of the genome. 35.5% of the contigs were longer than 2 kb and 5% of the contigs were longer than 5 kb. They used the EULER consensus generator and just assembled paired end plasmid reads, to reduce the high risk of mis-assembly that exists with such incomplete and repeat rich data. They now plan to improve this tool and to take on a pilot project using *Caenorhabditis briggsae* data.

David Jaffe (Whitehead Institute, USA) described the Arachne whole genome shotgun assembler, which we have already reported on in the highlights of the CSHL Genome Sequencing and Biology meeting in issue 2(4) of *Comparative and Functional Genomics*.

Colin Semple (University of Edinburgh, UK) presented **a computational comparison of human genomic sequence assemblies for a region of chromosome 4**. His group has a contig of 58 BAC/PAC clones covering a 5.8 Mb region linked to manic depression. These have been used to order 107 STS markers across the region. They used this high-resolution physical mapping data to assess the coverage of the region by the UCSC, NCBI and Celera genome assemblies. The principal observation was that there was significant variation between the three assemblies. In terms of the amount of sequence data assigned to the region, the NCBI had the highest coverage, at ~7 Mb, with UCSC having 6 Mb and Celera only 3.5 Mb. The STS ordering also varied markedly, with each assembly including different mis-assemblies. The Celera assembly had the least mis-assemblies, at 2 per Mb, and, interestingly, ~6% of its data was not represented in the public databases.

Shaying Zhao (TIGR, USA) described **a study of primate genome evolution using BAC end sequences**. They have generated paired end sequences from randomly selected BAC clones and BAC clones targeted to human hypervariable regions, from the chimp, baboon and lemur genomes. Using these sequences they have mapped the BACs onto the human genome, in an attempt to study long-range variations, such as deletions. The chimp and baboon sequences show higher average identities (97 and 95.6%, and 91 and 88.6%) compared to the lemur ones (80.9%, random only). Whereas 89–92% of baboon and chimp end pairs could be located, only 40% of lemur end pairs could be mapped. Their study shows that unique regions of the human genome tend to be more stable over evolutionary time than hypervariable regions, which show insertions, deletions and translocations.

Ingo Ebersberger (Max-Planck Institute for Evolutionary Biology) spoke about **a comparison of the chimp and human genomes**, which revealed a complex pattern of DNA sequence evolution. They

Copyright © 2001 John Wiley & Sons, Ltd.

have sequenced over 10 000 random clones from a chimpanzee shotgun library, representing some 3 Mb of the genome. They observe an average sequence difference (across the genome) of 1.26%. The X chromosome is the most conserved, as expected, while Y is the least conserved. They have looked at types of mutations, transitions at CpG, transitions at non-CpG, and the various types of transversions. The amount of sequence difference varies on the different autosomes, and transitions at CpG sites and A< >T transversions display different distribution patterns. They conclude that there are several factors that differ between autosomes, which influence the rates with which substitutions occur, and that these are broadly conserved in the human and chimp genomes.

Jo Dicks (John Innes Centre, UK) presented **CHROMTREE – A tool for deducing evolutionary histories using large-scale genomic data**. This tool looks at chromosomal evolution, by comparing gene order data between taxa and taking into account a selection of inherited events occuring within and between chromosomes, such as inversions, translocations, centric fusions and dissociations. The 'path' between any two chromosomes is defined as the sequence of events needed to get from one state to the other. The 'Pathloop' tool identifies all possible paths between two chromosomes by making one change and then trying out all possible events after that. The complexity of the tree that results is reduced (known as pruning) by discarding all those paths that are not making progress towards the target. Next she uses either an iterative segment model or an exponential failure model to determine the probabilities of the paths. Maximum likelihood and pairwise methods are then used to estimate phylogenies based on these data.

Joel Bader (Curagen Corp. USA) described their strategies for making **whole genome association studies with SNPs** financially viable. They plan to use only those SNPs that change amino acids in pharmacologically related genes, they estimate that there are ~10 000 of these markers in the 2–5000 genes of interest. To test just these in a population of 10 000 people, checking for 100 phenotypes per person would still cost around 10 million US dollars. To reduce this cost, they plan to make pools of patients with extreme phenotypes and look for SNPs that are enriched or depleted in these populations. They also plan to use linkage disequilibrium (LD) data to reduce the number of SNPs needed to cover the genome and aim to do both SNP and haplotype analyses.

### Gene prediction

Hugues Roest-Crollius (Genoscope, France) reported on the latest results in the project to sequence **the genome of the fish Tetraodon nigroviridis**, and its comparison to the human genome. On exceeding two genome equivalents of sequence the team have rerun their comparison against the Golden Path human genome assembly. Using their Exofish tool to detect 'ecores' (regions of conservation with the human genome) has proved a successful approach to detecting human exons. With their current data, they detect 122 000 ecores in the human genome, with an average of 50 per Mb. 53 109 of the ecores have matches in Refseq, hitting 10 454 of the 13 751 genes present. Using these matches they can estimate the number of ecores per human gene (3.86), and the number of human genes (31 000), although they do admit that Refseq does not represent a random sampling of human genes. They are now collaborating with the Whitehead Institute team, who have sequenced 2.25 genome equivalents of this genome.

Twinscan is a reimplementation of Genscan with an extended probability model. Paul Flicek (Washington University, USA) described this gene prediction tool, which we have previously reported on in the highlights of the CSHL Genome Sequencing and Biology meeting in issue 2(4) of *Comparative and Functional Genomics*.

Mark Yandell (Celera, USA) described the modifications made to Celera's 'ComputeCrawler' automated human genome annotation and analysis pipeline that have been made for the **annotation of mouse genome data**. There is a good numerical agreement between their *ab-initio* gene predictions for the two genomes and the numbers of high confidence annotations assigned by their tool 'Otto' are close. Comparisons of the predictions show that orthologs can easily be found in the majority of cases, but also indicate that there may only be as few as 20 000 orthologous human-mouse gene pairs.

Chris Southan (Gemini-Genomics, UK) presented a detailed example of how **chimeric mRNAs can result in annotation anomalies**. In the case of his proteins of interest, he observed ESTs from the two paralogues he had identified being placed into one transcript cluster, and erroneous clustering and

Copyright © 2001 John Wiley & Sons, Ltd. 

annotation of the proteins across three prominent databases, due to matches with chimeric mRNAs.

Thomas Down (Sanger Centre, UK) described a novel hybrid machine learning approach to **transcription start site (TSS) recognition**. They trained their TSS models (which use DNA weight matrices) using 263 mammalian promoters from the EPD database. They include elements such as the TATA box and GC rich motifs flanking the TATA box. To test the performance of the models, they used the chromosome 22 sequence. In comparing his tool against two others, he found that the vast majority of sites were found by all three, but that there were 85 which were not found by any of them. His tool predicted promoters for 46% of the transcripts, which were on average $\pm$ 10 bp from the true locations (where known), and the presence of CpG islands had little effect on his prediction rate.

Jean Thierry-Mieg (NCBI, USA) presented **AceView, a tool that defines gene structure using cDNA and EST data**. AceView aligns partial or complete mRNA sequences onto the genome. If an ORF exists, it will use 'tunneling' to try to extend exons. It selects only the best matches and allows cDNA mutations to be flagged, and sequencing traces to be edited. The tool can identify problem cDNA clones, such as those with chimeric inserts, partial or completely inverted inserts or deleted inserts. It can also recognises alternatively spliced transcripts, and will deduce an expression profile, based on the origin and abundance of the contributing clones. They are using AceView to annotate nematode genes and human genes and estimate that over half of the genes of each organism are currently represented in cDNA libraries. They have currently constructed 10 400 *C. elegans* genes, encoding 13 000 proteins and 30 000 human genes, encoding 90 000 proteins.

### Functional genomics

Anton Enright (EBI, UK) described an approach to **finding protein families in the draft human genome using Markov clustering**. This purely probabilistic, automated approach can accurately assign families based only on sequence similarity, without knowledge of domains. The sequence similarities are represented as a graph, where the nodes are proteins and the edges are the weighted similarity scores between them. Their algorithm calculates random walks through the graph, and models flow through the graph, until equilibrium is reached. Flow within a family of related proteins is higher than between families (as caused by a shared, promiscuous, domain). The clustering is calculated based on the flow through the graph. When the algorithm was tested using the InterPro, SCOP and SwissProt databases, it gave highly accurate clustering. The tool was recently used on the draft human genome for Ensembl, clustering 100 000 proteins into 13 000 families in just over six hours, on a small workstation.

Proteins can be also assigned to families based upon known structures. Julian Gough (MRC LMB, UK) described **a hidden Markov model approach to assigning sequences to families of known structure**. He used the SCOP database to generate a library of 4894 Markov models, called SUPERFAMILY, which represents essentially all proteins of known structure. The library has been run against over 50 complete genomes, matching twice as many target sequences as sequence similarity methods (many hypothetical proteins were found to be homologous to proteins of known structure). The coverage of the assignments was 35% for eukaryotic genomes and 43% for prokaryotic genomes. The annotations are available at **http://stash.mrc-lmb.cam.ac.uk/SUPERFAMILY**, where users can also match their own sequences against the library.

Hugh Salamon (Berlex Biosciences) has used **hidden Markov models for the prediction of experimentally verifiable protein functions**. The intention here is to discriminate between members of a protein family that do not possess a particular, measurable property, such as specific ligand binding, or signalling, and those that do. For this they require two sets of training sequences from which to derive their models, a set of proteins shown to have function of interest (a subfamily) and a set from the same family, without that ability (a different subfamily). Then they develop two sequence-weighted models against which any novel sequences are matched. This approach is currently being used to predict chemokine-chemokine receptor interactions.

Lue Ping Zhao (FHCRC, US) proposed **a statistical modelling approach for searching microarray datasets for genes showing stimulus-response**. In this case the focus is the study of the cell cycle. A simple model would be that a cell cycle gene would show repeatable cycling, however, in experiments, the cycling deteriorates after the initial release from the block used to obtain synchronisation of the cells. He has generated an alternative model of cell cycle responsive expression called the single pulse

Copyright © 2001 John Wiley & Sons, Ltd. 

model. His equation includes the basal expression level, the elevated expression level, the times of induction and return to basal level, and the time between the peaks of expression (or the cycle time). In testing on three budding yeast datasets, he detected 81% of the known periodic genes. 1088 genes showed periodicity in at least one of the three datasets, but only a quarter of these showed significant oscillation in two or more datasets and so can be classified with high confidence.

Steven Jones (GSC, Canada) presented **bioinformatic approaches developed for SAGE expression data**. Their software is designed to combine gene predictions from genomic data with EST data to allow the reconstruction of conceptual cDNAs including the untranslated regions (UTRs) required for unambiguous assignment of SAGE tags to transcripts. They also provide automated evaluation of libraries, which includes assessments of clone insert size, sequence quality and tag frequencies. The base pair qualities provided by PHRED, can later be used to discount tags with high error probabilities. They have also built a MySQL database for linking the expression data they generate with data from other resources, such as OMIM and dbEST. A Java-based expression viewer has been built to allow navigation of expression profiles in 3D space, it also allows for highlighting of genes of interest.

### Curation and ontologies

Hidemasa Bono (RIKEN, Japan) started off the session by reporting the current **status of the RIKEN Mouse cDNA Project**. Following the success of the FANTOM (Functional Annotation of Mouse) meeting in August 2000, where 21076 RIKEN clones were functionally annotated by a group of mouse genomics and bioinformatics experts, the team has produced an interactive viewer so that the annotation can be browsed from the web (**http://www.gsc.riken.go.jp/e/FANTOM/**). Preparations are being made for a follow-up meeting, FANTOM II, in November. This is to incorporate READ (RIKEN Expression Array Data) from adult and embryonic mouse tissues, along with protein interaction data from yeast two-hybrid assays and chromosomal mapping data of the cDNA clones.

Owen White (TIGR, USA) described the **features of the Comprehensive Microbial Resource (CMR)** that TIGR have made publicly available for scientists interested in microbial genomes. A team of eight curators manually annotate up to 200 genes per day for CMR. TIGRFAMS, a collection of protein families featuring curated multiple sequence alignments, support the automated functional identification of proteins by sequence homology. The interface to the CMR database allows the user to view a wide range of data fields, from specific genes, to vaccine targets. Also the user can examine different evidence of protein families to traverse across bacterial genomes.

The rat can be more useful than the mouse at modelling human disease. The annotation of this important mammalian genome is the aim of the **Rat Genome Database (RGD)** team. Simon Twigger (Medical College of Wisconsin, USA) detailed their efforts to integrate diverse rat genetic and genomic data by manual curation and automated means. Over 150 qualitative trait loci have been mapped to a genomic region, and using VCMap (Virtual Comparative Map), a dynamic sequence-based comparative tool, these regions can also be related to regions in mouse and human. Reflecting this cross-organism approach, RGD have developed a nomenclature pipeline to standardise nomenclature between Locuslink, Mouse Genome Database and Ratmap. In addition, they have adopted the standard vocabulary from the Gene Ontology consortium, to further facilitate common links between the three databases.

Tatiana Tatusova (NCBI, USA) presented the **genome resources at NCBI** and discussed their move from a two-week release of updates to overnight updating of genomic data. She presented some of the new features at NCBI, including BLink (BLAST Link), which displays the graphical output of pre-computed BLASTp; giving the user various options for displaying the information. In addition, she described the Entrez Map Viewer, which gives an integrated view of various genomes from different organisms so that map markers are linked directly to sequence data from Genbank entries.

The publication of the *Arabidopsis thaliana* genome marked a milestone in plant research. Heiko Schoof (MIPS, Germany) described the move for the **MIPS Arabidopsis thaliana database (MAtDB)** to integrate sequence data with experimental data, such as metabolic and phenotypic observations. As with any genomic database, updates are important, and MAtDB uses extrinsic data such as ESTs to correct gene predictions. It also allows external experts to edit information within the MAtDB

Copyright © 2001 John Wiley & Sons, Ltd. 

using specified ontology. Pedant, an automated protein annotation tool, is used alongside manual curation to give in-depth information on individual genes. They have also introduced BioRS, an integrated biological search and retrieval system, allowing complex queries across multiple databases for data-mining.

Joel Richardson (Jackson Laboratory, USA) discussed the **Mouse Genome Informatics** (MGI) group's integration of high-throughput automated genome analysis with manual, expert curation processes. The mouse research community was involved in producing standards for the Mouse Genome Database (MGD), such as nomenclature and a controlled vocabulary. Over 24 000 genes have been curated in the database. MGI members collaborated with the RIKEN team at an early stage, to produce a complete annotated mouse transcriptome, resulting in the first FANTOM meeting. This has been an example of the success that can be achieved from communication, co-ordination and co-operation within the mouse community. Another challenge the MGI group is tackling is to incorporate more biological data, such as that generated from microarray experiments and large-scale phenotyping or mutagenesis studies, into their database.

The final speaker in this session was Midori Harris (EBI, UK) from the **Gene Ontology (GO) Project**. She described the goal of the GO project: to provide a dynamic, controlled vocabulary, which can be applied to all organisms. At the moment, the vocabulary is subdivided into three key aspects of biology i.e. 'molecular function', 'biological process' and 'cellular function'. The GO consortium databases are currently using GO terms to provide high-quality annotation to genes and enable cross-organism searches based on GO annotation. The GO project is also involved in the development of software for querying, displaying and editing ontologies and associated gene products annotated with GO terms.

### Genome visualisation

Lincoln Stein (Cold Spring Harbor Laboratory, USA) began the session with a brief overview of the Gramene GO project.

Matthew Pocock (Sanger Centre, UK) continued with a detailed explanation of **how to build a distributed annotation system (DAS)**. Such a system is able to collate data over the Internet from many different data sources, pool them according to a common co-ordinate system and display the result on the user's computer in a simple graphical format. At present, such a system is able to analyse sequence annotation data but many of the audience noted that the system could be extended for other data types. The three-layer system is topped by a simple Java client application for browsing the DAS system. The authors encourage others to set up DAS servers for their own databases.

John Quackenbush (TIGR, USA) described a series of tools his group has developed for the **analysis of microarray gene expression data**. He began by outlining the Minimal Information for a Microarray Experiment (MIAME) project, which has produced a standard for microarray data representation. Two leading corresponding XML formats submitted to the Object Management Group (OMG), MAML and GEML, were recently merged into a single format, MAGEML. This means that there will be a single standard format for this type of data, for bioinformaticians everywhere to use, if they wish, making it much simpler to pool data from different experiments and to interface to publicly available tools. John outlined the TIGR data pipeline for the analysis of microarray data. This pipeline begins with the TIGR Gene Index (TGI) which links via the RESOURCER database and the MADAM Microarray Data Management system to the Multiple Experiment Viewer (MeV), which allows simultaneous visualisation and analysis of a collection of gene expression experiments.

The next speaker, Toshihiko Honkura (University of Tokyo, Japan), showed a novel approach to displaying a very large quantity of WWW data within a single graphical representation, with his **GRL_VIEWER for gene structures and splicing patterns**. His browser, based on the Macromedia Flash technology, enabled low-bandwidth dynamic representations of millions of EST locations and structures to be displayed alongside chromosomal regions and a variety of genomic sequence annotations. The Gene Resource Locator homepage (**http://grl.gi.k.u-tokyo.ac.jp/**) provides a query interface for users to browse rapidly for alignments of genes of interest.

Mark Wilkinson (Plant Biotechnology Institute, USA) gave an overview of the **Genquire system for fast, interactive genome browsing and annotation**. Mark described the three layers of the Genquire system, the first being the 'Genome' level where

Copyright © 2001 John Wiley & Sons, Ltd.

users can see a clickable display of the chromosomes. This level interfaces to various tools, such as BLAST, and leads to the 'Conti' level. This level displays sequences stacked according to source origin and may be analysed by the BLAST or Sim4 programs. Finally, a 'Nucleotide' display of sequence data allows highlighting of interesting and unusual sequence features. Genquire includes a Gene Ontology browser and annotation tools. Potential users were encouraged to code their own simple adapters for non-Genquire databases, so that they could use the Genquire system.

Eluemuno Blyden (LabBook Inc., USA) introduced the **Bioinformatics Sequence Markup Language (BSML)**. He described the growing recognition of the importance of the Extensible Markup Language (XML) in bioinformatics, allowing for the exchange of domain specific knowledge. He went on to explain that BSML is a public protocol based on XML and currently allowed for data annotation and display. He described the Genomic XML Viewer®, a publicly available BSML browser (**http://www.Labbook.com**) that provides interactive displays for the visualisation of sequences and annotations from local and WWW data sources.

Guy Davenport (John Innes Centre, UK) described his **ARCADE system for comparative genome analysis** via the collation and display of comparative mapping data. ARCADE, developed for the UK CropNet project (**http://ukcrop.net/**), enables complex comparative queries to be made on a series of single-species or related-species databases, linked by one or more comparative mapping databases. Query result sets can be displayed on a series of custom Java comparative viewers. ARCADE is a Java/XML system that interfaces to over 20 plant ACEDB databases and will, in the future, communicate with non-ACEDB databases.

The final talk in this session was given by Kim Rutherford (Sanger Centre, UK), who described **Artemis, a Java tool for the pairwise comparison of whole genomes**. Artemis, which shows a very detailed view of the DNA sequences of two genomes, takes its data in EMBL, Genbank or GFF formats. Pairwise comparisons are generated from tools such as BLAST. The tool displays such features as syntenic regions, insertions, deletions, and repeats. It is currently being used for the analysis of genomes sequenced at the Pathogen Sequencing Unit, but it was hoped that it could also be used for the analysis of larger genomes.

---

The Meetings Highlights of *Comparative and Functional Genomics* aim to present a commentary on the topical issues in genomics studies presented at a conference. The Meetings Highlights are invited and each represents a personal critical analysis of the current reports and aim at providing implications for future genomics studies.

Copyright © 2001 John Wiley & Sons, Ltd.

